# Non-communicable Diseases and Cognitive Impairment: Pathways and Shared Behavioral Risk Factors Among Older Chinese

**DOI:** 10.3389/fpubh.2019.00296

**Published:** 2019-10-23

**Authors:** Vasoontara Sbirakos Yiengprugsawan, Colette Joy Browning

**Affiliations:** ^1^Centre for Research on Ageing, Health and Wellbeing (CRAHW), Research School of Population Health, The Australian National University, Canberra, ACT, Australia; ^2^Australian Research Council Centre of Excellence in Population Ageing Research (CEPAR), University of New South Wales, Kensington, NSW, Australia; ^3^School of Nursing and Healthcare Professions, Federation University, Ballarat, VIC, Australia; ^4^International Primary Health Care Research Institute, Shenzhen, China

**Keywords:** aging, China, cognitive decline, chronic conditions, lifestyle risk factors

## Abstract

Population aging has brought about a number of challenges to public health and primary health care systems due to increases in the prevalence of non-communicable diseases (NCDs). As a country with one of the largest populations globally, China is confronting a rising number of chronic NCDs including cardiometabolic related conditions. This mini-review investigates the link between NCDs and cognitive impairment through common risk factors. Identifying risk factors is important for the prevention and management of these chronic conditions. In addition, this review also identifies the role of primary health care services in reducing behavioral risk factors for NCDs and cognitive impairment. Addressing shared determinants and pathways is important in the design of public health interventions and primary health care services in China. Monitoring and management of NCD biomarkers and behavioral risk factors may also be beneficial for cognitive health among older Chinese.

## Introduction

As China's population ages, the country is confronted with rapidly changing health profiles. According to the Global Burden of Diseases, the pattern of causes of deaths has also changed significantly away from communicable diseases and as of 2016, the main causes of death were cardiovascular diseases (41%), cancers (25%), chronic respiratory diseases (10%), and diabetes (4%) (1). In addition to these “Big 4” non-communicable diseases (NCDs), neurological disorders including Alzheimer's disease and other dementias further contributed to 5% of pre-mature mortality. This figure is expected to increase as China's population ages (1, 2).

NCDs are inextricably linked to natural aging processes and various modifiable risk factors, and as a result, chronic conditions generally have common determinants and many shared risk factors, most of which are amenable to prevention and amelioration over the lifecourse (3, 4). NCDs have direct or indirect impacts on activities of daily living resulting from these conditions (5). Furthermore, there has been emerging literature on the potential links between chronic non-communicable diseases and cognitive impairment, notably highlighting common mid-life modifiable risk factors (e.g., mid-life hypertension, diabetes, smoking), improving integrated chronic care management, and reducing impacts on overall quality of life (6–8).

The United Nations Sustainable Development Goals initiative has called for a reduction by one third of premature mortality from NCDs (9). If this is to be achieved in China, risk factor reduction needs to be a focus. An analysis of Chinese Burden of Disease risk factor data (blood pressure, body mass index, high cholesterol, high fasting glucose, smoking, and inactivity) in people aged 30–70 years, found that if risk reduction targets were met by 2030, one million deaths could be avoided (10).

While there is good evidence that the prevalence of cognitive impairment among older Chinese is greater than would be expected from age-related health decline (2, 11, 12), information is still limited on the relationships between NCD co-morbidities, common risk factors, and cognitive impairment. This review looks at common pathways for NCDs, Cognitive Impairment (CI), and Mild Cognitive Impairment (MCI) whereby CI is a broad term that can range from mild to severe (13). The purpose of this review is to specifically focus on older Chinese to investigate associations between NCDs, CI as well as MCI, and common risk factors, as well as to identify the role of primary health care services in reducing common behavioral risk factors. Notably, the majority of these studies reported focus on MCI with some examples of CI included. The role of key socio-demographic factors and primary health care in these associations will be also presented in the mini-review.

The review will contribute to a better understanding of the pathways and interrelationships between NCDs, CI/MCI and their risk factors among older Chinese. It will also discuss effective health promotion and prevention strategies targeting modifiable risk factors that may prevent or delay the onset and progress of late-life cognitive impairment.

## Methods

This mini review will cover the range of observational population-based studies conducted between 2010 and 2018 focusing primarily on CI/MCI among non-clinical samples of adults aged 50 years and over in mainland China. Studies that restricted the sample ages to 75+ years and the oldest old were not included in the review. Main searches were conducted in English through international databases such as MEDLINE, PubMed, Scopus, and Web of Science. Key search terms, for example, include “cognitive impairment” “Mild Cognitive Impairment” “MCI” “Alzheimer's” “dementia” “cardiovascular diseases” “hypertension” “diabetes” “blood pressure” “chronic diseases” (Table 1).

**Table 1:** Description of studies illustrating relationships between cognitive impairment and non-communicable diseases.

**Studies**	**N and age (years)**	**Areas**	**Study design**	**Cognitive impairment (CI/MCI)**	**Cardiometabolic and lifestyle factors**
Gao et al. (14)	*N* = 8213 Aged 65+	Tianjin, Northern China	Cross-sectional (2010)	Mini Mental State Examination MMSE ≤27 Montreal Cognitive Assessment MoCA <26	Fasting plasma glucose (FPG), glycosylated hemoglobin (HbA1c) and immunoreactive insulin (IRI) were associated with increasing risk for MCI with Type 2 diabetes (T2DM).
Li et al. (15)	*N* = 525 Aged 55+	Shanghai	Cross-sectional (2011)	MMSE ≤27 MoCA <26	T2DM was a risk factor for MCI progressive to Alzheimer's disease.
Wang et al. (16)	*N* = 480 Aged 65+	Tianjin, Northern China	Longitudinal (2008–2014)	MMSE <20 (primary education); MMSE <24 (>primary education) MoCA <25 (≤12 years of education); MoCA <26 (>12 years of education)	Being overweight or obese at baseline was associated with an increased risk of both amnestic and non-amnestic MCI. An increased body mass index at 6-year follow-up also increased the risk of non-amnestic MCI.
Li et al. (17)	*N* = 865 Aged 55+	Xi'an	Cross-sectional (2014/2015)	MMSE <17 (illiterate) ≤20 (primary school education) ≤24 (junior school and above)	Significant interaction between abdominal obesity and diabetes associating with an increased risk of cognitive impairment.
Wu et al. (18)	*N* = 2,065 Aged 60+	Wanshoulu District, Beijing	Cross-sectional (2009/2010)	MMSE <17 (illiterate) ≤20 (1–6 years of education) ≤24 (≥7 years of education)	Prevalence of MCI was higher in hypertensive than normal individuals. Among hypertensive patients, the prevalence of MCI was lower in those treated than in those not treated.
Ren et al. (19)	*N* = 1,171 Aged 60+	Tianjin, Northern China	Cross-sectional (2014/2015)	MMSE <17 (illiterate) <22 (primary school) <26 (junior school and above)	In the multivariate analysis, high blood pressure/ stage III hypertension was associated with cognitive impairment.
He et al. (20)	*N* = 227 Aged 65+	Tianjin, China	Cross-sectional (2014)	MMSE scores 18–23: mild CI MMSE scores 10–17: moderate CI MMSE scores 0–9: severe CI	Total cholesterol (TC) was significantly higher in participants with MCI. Elevated HDL (high-density lipoprotein cholesterol) and triglyceride were associated with the occurrence of MCI.
Ma et al. (21)	*N* = 1,159 Aged 60+	Nationwide, Chinese Longitudinal Healthy Longevity Survey	Longitudinal (2009–2014)	Annual cognitive change of MMSE scores	High TC and low-density lipoprotein cholesterol (LDL-C) in late-life were associated with greater cognitive decline.
Zou et al. (22)	*N* = 597 Aged 60+	Chongqing, Southwest China	Cross-sectional (2011/2012)	Complaint of memory decline for at least 6 months; objective memory decline via neuropsychological evaluation, Clinical Dementia Rating (CDR) score of 0.5.	Hypertension, coronary heart disease, TC, and low-density lipoprotein cholesterol (LDL-C) are independent risk factors for MC.
Yin et al. (23)	*N* = 16,629 Age 60+	Nationwide, Disease Surveillance Point System	Cross-sectional (2011/2012)	MMSE <17 (illiterate) ≤20 (primary school) ≤24 (middle school and above)	Chronic respiratory symptoms and self-reported COPD were strongly associated with cognitive impairment in urban areas.
Zhou et al. (24)	*N* = 3,012 Age 60+	Chongqing, Southwest China	Cross-sectional (2001)	MMSE <17 (illiterate) <20 (primary school) ≤24 (middle school and above)	Current smoking and daily alcohol consumption were significantly associated with an increased risk of cognitive impairment.
Pan et al. (25)	*N* = 2,037 Age 45+ women only	China Health and Retirement Longitudinal Study	Longitudinal (2011–2013)	Telephone Interview version of the MMSE – 1) memory tests and 2) orientation, visuoconstruction, and numeric ability.	Longer exposure to second hand smoke exposure was associated with a greater decline in memory over 2 years, especially among women aged 55–64 years
Zhu et al. (26)	*N* = 6,586 Age 65+	Chinese Longitudinal Healthy Longevity Survey	Longitudinal (2005 and 2008–2009)	MMSE <18 (no formal education) <21 (1–6 years of education) <25 (>6 years of education)	High level of participation in leisure activities was associated with about 40% decreased risk of cognitive impairment.

### Definition and Prevalence of CI/MCI Among Older People in China

We focus on CI and MCI as there is evidence that these conditions could lead to an increased risk of developing more severe neurological conditions such as dementia and Alzheimer's disease (2, 11, 12). Commonly used international diagnostic criteria for CI and MCI rely on cognitive function assessments such as the Montreal Cognitive Assessment (MoCA) and the Mini-Mental State Examination (MMSE), both with scores ranging from 0 to 30 points. MCI diagnosis is often based on Petersen's criteria which include memory problems in the absence of dementia (27–29). In the Chinese literature, using MMSE, CI has a cut-off score of 24, while MCI generally has the cut-off score of 26 (30–32). Taking into account the educational differential effect on cognitive performance, MCI among older Chinese was commonly defined by an MMSE score of <17 in the illiterate group, <22 in the primary school group, and <26 in the junior school and above (19). Corresponding cut-off scores for CI were reported at 17, 20, and 24, respectively (23).

According to a meta-analysis of 22 studies among older people aged 60 years and over in China, 12.7% have experienced MCI and the prevalence was lower in more economically advanced Eastern areas (9.6%) compared to Western China (14.7%) (11). A more recent systematic review among 48 studies across 22 provinces in China reported the pooled prevalence of MCI in the older Chinese aged 60 years and over was 14.7%; the prevalence was 16.7% in clinical samples and 14.6% in non-clinical samples (12).

## Results

### Relationships Between Cardio-Metabolic Risk Factors and CI/MCI

#### Diabetes and Obesity

There is increasing evidence on the significant relationship between type 2 diabetes mellitus and CI/MCI among older adults in China (14, 15, 33). A cross-sectional study (aged 65+in Northern China) revealed risk factors for MCI to be age at onset and biomarkers of type 2 diabetes severity such as fasting plasma glucose, glycosylated hemoglobin, and immunoreactive insulin (14). An urban population-based study (aged 55+ in Shanghai) further confirmed that T2DM was not only a risk factor for MCI but also associated with the progression to Alzheimer's disease after adjusting for other possible covariates (15). However, another study (aged 60+ in Tianjin) revealed that even in the absence of type 2 diabetes, being overweight and/or obese are risk factors for MCI (16). Similarly a community-based study (aged 55+ in rural Xian) found that the presence of both type 2 diabetes and abdominal obesity was associated with an increased risk of cognitive impairment by more than double (17).

#### Cardiovascular Diseases, Blood Pressure, and Cholesterol

Cardiovascular diseases and cognitive decline share common risk factors including high blood pressure, which was found to be a predictor for CI/MCI among older adults (32, 34). A cross-sectional study (aged 60+ in Beijing) reported a higher prevalence of MCI among hypertensive individuals and also the prevalence of MCI was lower in those treated than in those not treated (18). Another cross-sectional study (aged 60+ in Tianjin) also reported similar findings with high blood pressure associated with a higher risk of cognitive impairment compared to normal blood pressure participants (95% CI: 1.07–2.54) (19). In addition to hypertension, coronary heart disease, total cholesterol, and low-density lipoprotein cholesterol were found to be independent risk factors for MCI and elevated high-density lipoprotein cholesterol and triglyceride were associated with the occurrence of MCI (20–22).

#### Chronic Obstructive Pulmonary Disease

There is evidence documenting the relationships between chronic obstructive pulmonary disease and cognitive impairment among older Chinese. A nationally representative Chronic Disease and Risk Factor Surveillance study covering 31 provinces across China reported chronic cough, chronic phlegm, and self-reported symptoms in urban areas, but only chronic phlegm in rural areas was associated with cognitive impairment (23). In addition, indoor air pollution, such as exposure to biomass combustions from cooking fuel, could modify the effects of chronic obstructive pulmonary disease on cognitive impairment (23).

### Relationships Between Modifiable Health-Risk Factors and Cognitive Impairment

#### Smoking and Alcohol Consumption

Smoking and alcohol consumption have been found to be major behavioral risk factors for cognitive impairment among older Chinese (24, 25). For example, cross-sectional evidence from (aged 60+ in Chongqing) reported that current smoking and daily consumption of alcohol were associated with a significantly increased risk of cognitive impairment (24). Despite of low female smoking in China, further longitudinal evidence from the national China Health and Retirement Longitudinal Study among Chinese nationwide reported that longer exposure to second hand smoke was associated with higher risk of cognitive decline over 2 years, especially among women aged 55–64 years (25).

#### Exercise and Incidental Physical Activity

The benefits of being physically active has been widely documented among older Chinese with direct effects across multiple domains of cognitive functions from engaging in modern aerobic and traditional Chinese exercise such as Tai Chi and Qigong (35–37). A systematic review among older Chinese has linked the health benefits of exercise in a range of chronic diseases through lowering elevated blood pressure (hypertension) as well as improving cardiopulmonary and lung functions (38). In addition to exercise, incidental physical or leisure activities such as housework were shown to associate with a 41% decreased risk and thus protective against cognitive impairment based on the 5-year follow-up of the nationwide Chinese Longitudinal Health Longevity Survey (26).

### Common Social Determinants of NCDs and Cognitive Impairment

#### Socio-Economic Status

Low socioeconomic status has been commonly linked to disparity in mild cognitive impairment (11, 19, 39). According to the World Health Organization Study on global AGEing and adult health among older Chinese nationally aged 50 years and over, years of education were shown to be significantly associated with cognition scores at the individual level and median household income and median years of education at the community level (40). Using the same data, another study has confirmed that both education level and wealth were negatively associated with untreated chronic non-communicable diseases among older Chinese (41).

#### Demographic Status

The role of demographic characteristics, particularly old age and being female is associated with cognitive impairment among older persons across China (11, 19). The gender difference may also be a result of women having less access to education (11, 42). Solitary status, including never married, divorced, and widowed, is also associated with cognitive impairment among older Chinese (43).

#### Geographical Areas

Multiple epidemiological studies have determined that geographical disparities result in an increase in cognitive impairment among older Chinese in rural areas (39, 44). Residential status has been linked to a faster decline in cognitive function, not only among rural but also rural-to-urban migrants compared to urban residents as reported by a 12-year follow-up of the Chinese Longitudinal Health Longevity Survey (45). Another study using a 9-year follow-up of the same data revealed rural disadvantage also affected not only cognitive health but also other physical health measures (46).

### Strengths and Limitations

This mini review brings together recent evidence on cognitive and mild cognitive impairment among older Chinese highlighting the link to shared behavioral lifestyle risk factors of cardio-metabolic conditions. The strength of this mini review draws upon many large epidemiological studies across China including longitudinal studies where possible. Notably, the mini review focuses on observational population-based data with less evidence from clinical studies. Because of the smaller scope of the mini review, which is primarily based on international databases, relevant articles in Chinese may be omitted. This could potentially result in under reporting bias in some geographical areas that have limited exposure to English publications.

The definition and severity of cognitive impairment vary across studies, however, most of these studies use international standardized measures. It has been argued that these measures should have differing cut-offs for older and less educated respondents within the Chinese context (47). Care should be taken in the investigation of aging and cultural influence on cognition (48, 49). In addition to the importance of culturally appropriate tests in interpreting cognitive impairment, other factors such as cultural specific factors such as regional norms among older Chinese could be further explored. Longitudinal studies could provide insight into mechanisms by which cognitive impairment and NCDs interact. Standardized measures would also allow for possible international comparative studies beyond China in future research.

### Policy Implications

Under the national Healthy China Initiative, the Chinese government has put in place the plan toward 2025 for the prevention and treatment of chronic non-communicable diseases (50). The first two goals of “…diagnosing diseases in the early stage” and “…advocate a healthy lifestyle” are applicable to both NCD and CI/MCI which share common risk factors. Addressing behavioral risk factors and early detection of NCDs could delay its progression and further complications.

Since the health system reforms in 2009, achieving essential public health services and community-based non-communicable disease management has been rolled out nationwide to improve availability and accessibility of health services (51). Primary health care and community health centers, especially in the rural areas, play a significant role in regular screening for blood pressure or blood glucose in middle-aged and older adults and follow-up management of these conditions (52, 53). In line with advocating a healthy lifestyle through self-care education, behavioral, and psychological approaches embedded within primary health care settings could also provide effective intervention in managing chronic conditions such as diabetes (54). Integration of services at both primary and secondary levels is also vital for chronic care management (5, 55).

In order to implement these reforms in primary health care settings the health care workforce needs to be upskilled (50, 56). The need for more and better trained general practitioners is central to many recent policy statements in China (57). Sun et al. (58) in this *Frontiers Special Topic Chronic Illness and Aging in China* present a strong argument for the training of general practitioners in China in behavior change and chronic illness management in order to prevent and manage behavioral risk factors in chronic illness. Wang et al. (57) in an analysis of community nurse training courses asked nurses to rank the importance and the utility of various topics. Pertinent to the current paper the authors found that while learning about health behaviors was ranked by nurses as 11 out of 20 topics in terms of importance, content utility was ranked at 15. In the subsequent modified curriculum, methods and skills involved in health education and promotion, impacts of unhealthy behaviors and interventions for unhealthy behaviors were included as well as management of chronic disease in the community.

The recent World Bank China Joint Study Partnership Health Policy Summary “Deepening health reform in China” (56) advocates a People-Centered Integrated Care Model to address the burden of chronic illness in China. It proposes an expanded and empowered role for citizens in the management of their health through building health literacy, strengthening patient self-management, and improving shared decision making. This model has the potential to address risk factors and the management of chronic illnesses in China but will require an investment in the training of doctors in the areas of self-management and behavior change and the education of citizens in the prevention of chronic illness.

As well as training the healthcare workforce in people-centered care, risk factor and chronic disease management; and the empowerment of patients in the management of their illness, system level changes are also needed. In addition to workforce strengthening and engaging citizens in their health care, the Deepening Health Reform in China policy statement identifies another six levers of reform including shaping a tiered health care delivery system, improving quality of care, reforming public hospitals, realigning purchaser and provider payment incentives; strengthening the health care private sector and modernizing health service planning [(56) p. xviii].

Given our current knowledge about the relationships between NCDs and cognitive impairment it is important that early public health interventions and primary health care services address risk factors across the lifecourse and that these interventions and services are well-targeted. We argue that attention to prevention through reducing behavioral risk factors such as excess tobacco and alcohol consumption and physical inactivity and the management of abnormal blood pressure and blood glucose all have the potential to impact positively on cognitive and mild cognitive impairment and non-communicable diseases in China.

## Author Contributions

VY and CB conceptualized the study, drafted the manuscript, and finalized revisions for publication.

### Conflict of Interest

The authors declare that the research was conducted in the absence of any commercial or financial relationships that could be construed as a potential conflict of interest.
